# Isolation and Characterization of Laccase from *Trichoderma asperellum* Tasjk65

**DOI:** 10.3390/biology14060691

**Published:** 2025-06-13

**Authors:** Kehe Fu, Lili Fan, Qi Li, Jiaming Ji, Zhenying Huang, Ting Huang

**Affiliations:** College of Life Science, Nanchang Normal University, Nanchang 330032, China; llfan31@163.com (L.F.); 15720905198@163.com (Q.L.); kelgi12367@163.com (J.J.); HZying211@163.com (Z.H.); Child1100@163.com (T.H.)

**Keywords:** *Trichoderma asperellum*, laccase, enzyme kinetics, gene knockout

## Abstract

Laccase catalyzes one-electron oxidation, producing water as the primary final product, thereby minimizing secondary environmental pollution. Consequently, it holds significant application potential in areas such as the degradation of toxic compounds. In this study, a high-laccase-producing *Trichoderma* strain was isolated from soil, and the conditions for laccase production were optimized. Additionally, the laccase-related gene was cloned, and its function was analyzed. The results revealed that the optimal conditions for laccase production in this strain were maltose as the carbon source, peptone as the nitrogen source, an optimal pH of 6.0, and an incubation time of 120 h, resulting in an enzyme activity of 1.32 U/mL. The purified enzyme exhibited a Michaelis constant (*K_m_*) of 0.06666 mmol/L when ABTS was used as the substrate. SDS-PAGE analysis indicated that the enzyme’s molecular weight was approximately 70 kDa. Sequencing of the target protein band led to the identification of the laccase-related gene *Tasla01*. Knockout of this gene resulted in the loss of laccase activity. We isolated a high-laccase-producing *Trichoderma asperellum* strain, Tasjk65, and cloned the laccase-related functional gene *Tasla01*. These findings lay a foundation for the source and application of laccase.

## Simple Summary

Environmental pollution caused by organic compounds remains a significant challenge. Laccase enzymes are widely utilized for pollutant degradation due to their high efficiency, cost-effectiveness, and lack of secondary pollution. In this study, we isolated a high-laccase-producing strain of the fungus *Trichoderma*, designated Tasjk65, which achieved an optimized enzyme activity of 1.32 U/mL. The laccase demonstrated substantial degradation of organic dyes. Furthermore, we cloned and characterized the functional genes associated with the laccase. These results lay a foundation for the future application of laccase enzymes in environmental remediation.

## 1. Introduction

In recent years, the significance of enzymes in industrial applications has become increasingly pronounced. These biocatalysts are extensively utilized in various industrial processes to facilitate cleaner, more efficient, and more sustainable production methods [1]. Laccase, an ancient and extensively studied enzyme, is widely distributed in nature [2]. Laccase (benzenediol: oxygen oxidoreductase, EC1.10.3.2) is a metalloprotein within the multicopper oxidase (MCO) family, comprising three copper-binding domains [3]. The active site of laccase generally contains four copper ions, coordinated by the T1, T2, and T3 centers. Among these, the mononuclear T1 center exhibits the highest redox potential, enabling the oxidation of the substrate and electron transfer to the trinuclear center formed by the T2 and T3 centers, ultimately reducing oxygen to water [4]. Laccase catalyzes the oxidation of various compounds, including polyphenols, diamines, aromatic amines, and inorganic ions [5]. Its broad substrate specificity renders it highly valuable in applications such as lignin valorization, biopolymer degradation, bioremediation, and dye decolorization [6,7]. Laccase also has been used successfully to remove micropollutants from wastewater, as it has the ability to remove a wide range of pharmaceuticals, steroid hormones, personal care products, pesticides, and industrial chemicals [8]. To enhance utilization efficiency, extensive research has been devoted to the immobilization of laccases on solid supports. Zimmermann et al. demonstrated that the laccase from the white-rot fungus *Coriolopsis polyzona*, when immobilized on spherical nanoparticles, retained its activity in effluent water for over one month [9]. Laccase is predominantly found in plants, fungi, and bacteria, with a diverse range of biological sources, including bacteria, fungi, plants, and certain insects [10]. In 1993, Givaudan first identified bacterial laccase in nitrogen-fixing bacteria isolated from the rhizosphere soil of rice [11]. Bacterial laccase is present in various forms and exhibits a wide range of molecular weights, participating in multiple physiological processes. Plant laccase plays a crucial role in tissue regeneration following damage and collaborates with peroxidases to facilitate the synthesis of lignin polymers [12]. Notably, fungi are the primary producers of laccase, with the most extensive research conducted in this area. Fungal laccase exhibits a higher redox potential than bacterial and plant laccases, providing it with a clear advantage in bioremediation [13,14]. Research indicates that the redox potential of fungal laccase typically exceeds 400 mV, enabling the efficient decomposition and removal of various high-redox-potential contaminants, such as plastics, hydrocarbons (particularly polycyclic compounds), phenolic compounds, dyes, and other pharmaceutical substances [15,16,17]. These attributes confer substantial potential for the application of fungal laccase in environmental remediation, wastewater treatment, and organic pollutant degradation.

*Trichoderma* spp. are a group of filamentous fungi that are globally distributed and commonly found in natural environments, including soil, plants, plant residues, and the rhizosphere [18,19]. Due to their rapid growth, ease of cultivation, strong adaptability to diverse environments, and high enzyme production capabilities, these fungi have increasingly become a focal point in research and industrial applications related to fungal laccases [20]. Several strains of the *Trichoderma* genus demonstrate extracellular laccase activity, including *T. green*, *T. lignorum*, *T. reesei*, and *T. longibrachiatum* [21,22]. Moreover, *Trichoderma* spp. are recognized for their substantial capacity to produce active laccase, which positions them as environmentally friendly catalysts [23]. Furthermore, the laccases produced by *Trichoderma* demonstrate broad specificity for various substrates and possess high stability, indicating potential applications in fields such as wastewater treatment, dye decolorization, synthetic material modification, and biosensing. In a previous study, a codon-optimized laccase gene was used to construct a recombinant plasmid, which was then transformed into *T. lignorum*, enabling the efficient extracellular expression and secretion of exogenous laccase. The resulting laccase demonstrated significant decolorization of active brilliant blue KNR in textile wastewater, further highlighting its potential in dye decolorization [24]. Additionally, research by Sadhasivam et al. indicated that laccase from the *Trichoderma* strain WL1 displayed effective decolorization during the pulp bleaching process [25]. This enzyme successfully degraded lignin and harmful organic compounds present in paper mill wastewater, significantly reducing both the chemical oxygen demand (COD) and the color of the effluent. Furthermore, the incorporation of laccase in traditional bleaching processes minimizes the use of chlorinated compounds, thereby mitigating environmental pollution.

Despite advancements in the research of *Trichoderma* laccases, optimizing cultivation conditions and elucidating the enzyme’s biochemical properties remain significant challenges. These issues hinder the effective utilization of laccase as a biocatalyst, thereby limiting its broader industrial applications. To address this challenge, in the present study, a laccase-producing strain was isolated from soil samples collected in Nanchang City, Jiangxi Province. The primary objective was to enhance the laccase production yield of this strain through the optimization of its cultivation conditions. Furthermore, laccase purification was conducted using techniques such as ammonium sulfate precipitation, dialysis, and ion-exchange chromatography, in conjunction with an investigation of its enzymatic characteristics. Additionally, a protein sequencing analysis was employed to identify genes related to laccase production and to assess their functions, with the aim of elucidating the molecular mechanisms that underlie laccase production in this strain. The aim of this research was to provide a robust theoretical foundation for the industrial application of laccase.

## 2. Materials and Methods

### 2.1. Materials

*T. asperellum* Tasjk65 and the gene knockout plasmid are preserved at the Molecular Genetics Laboratory of Nanchang Normal University.

TSM (*Trichoderma* selective medium) (g/L, gram per liter): MgSO_4_∙7H_2_O 0.2, K_2_HPO_4_ 0.9, KCl 0.15, NH_4_NO_3_ 1.0, glucose 3.0, Rose Bengal 0.15, agar powder 15, water 1000 mL [26].

PDA (potato dextrose agar) (g/L): peel potatoes 200, anhydrous glucose 20, agar powder 15.

SNA (synthetic nutrient-poor agar) (g/L): KH_2_PO_4_ 1.0, KCl 0.5, KNO_3_ 1.0, MgSO_4_·7H_2_O 0.5, glucose 0.2, sucrose 0.2, agar powder 14, water 1000 mL [26].

Laccase basic fermentation medium (g/L): peptone 5, maltose 10, KH_2_PO_4_ 1, MgSO_4_·7H_2_O 0.5, CuSO_4_·5H_2_O 0.2, water 1000 mL, pH 6.0.

Czapek agar (CA) (g/L): KH_2_PO_4_ 1.0, NaNO_3_ 2.0, MgSO_4_·7H_2_O 0.5, KCl, 0.5 FeSO_4_·7H_2_O 0.01, sucrose 30, agar powder 15.

IM (induction medium) (g/L): KH_2_PO_4_ 1.45, K_2_HPO_4_ 2.05, NaCl 0.15, MgSO_4_·7H_2_O 0.5, CaCl_2_ 0.05, FeSO_4_·7H_2_O 0.0025, (NH_4_)_2_SO_4_ 0.5, glucose 0.72, agar powder 15 [27].

### 2.2. Screening of Laccase-Producing Strains

First, 10 g of soil was weighed and placed in an Erlenmeyer flask containing 90 mL of sterile water. After thorough shaking, a gradient dilution was performed up to 10^−3^. Subsequently, 200 µL of the diluted solution was spread on plates containing TSM medium (supplemented with 50 µg/mL streptomycin) and incubated upside down at 28 °C for 3 to 5 d until single colonies appeared. The single colonies were then transferred onto PDA medium containing 0.4% guaiacol and incubated at 28 °C for 48 h in the same upside-down orientation. A reddish-brown oxidative zone was observed around the colonies, and the strain was recorded for further screening. The enzyme production capabilities of the strains were further confirmed through shake flask screening. Three 0.5 cm diameter agar plugs from the colonies were inoculated into 250 mL Erlenmeyer flasks containing 50 mL of laccase fermentation medium, and the flasks were incubated at 28 °C, with shaking at 200 rpm for 5 d. The supernatant was obtained by filtering through a 0.22 µm filter, yielding a crude enzyme solution. Laccase activity was determined using the ABTS method, and the strain demonstrating the highest enzyme activity was selected for subsequent experiments.

### 2.3. Identification of Laccase-Producing Trichoderma spp.

Place a sterile coverslip onto the PDA plate and introduce agar plugs on the center of the plate. Incubate at 28 °C until the mycelium extends to the coverslip and conidia are produced. Subsequently, remove the coverslip and position it face down on a slide that contains a small amount of water in the center. Observe the sample under a microscope (MD50, Guangzhou Mingmei Technology Co., Ltd, Guangzhou, China.) while simultaneously recording the growth and morphological characteristics of the colonies. To assess the growth rate of the strains, inoculate the strains onto PDA and SNA plates and incubate at 28 °C. Measure the colony diameter every 12 h.

Genomic DNA from the target strain was extracted using the CTAB method [28]. Primers were designed to amplify translation elongation factor 1-alpha (*tef1*) [29]: tef1F: CATCGAGAAGTTCGAGAAGG; tef1R: AACTTGCAGGCAATGTGG. PCR amplification was conducted, and the products were analyzed using 0.1% agarose gel electrophoresis. Following purification, the samples were submitted to Shanghai Bioengineering Company for sequencing. A phylogenetic tree was constructed using the neighbor-joining (NJ) method implemented in Molecular Evolutionary Genetics Analysis version 7.0 (MEGA7) software [30].

### 2.4. Laccase Activity and Characterization

#### 2.4.1. Determination of Laccase Activity

To determine laccase activity, a blank control tube containing 2 mL of a citric acid–sodium hydrogen phosphate-buffer solution (pH 3.0) and 1 mL of a 1 mmol/L ABTS solution was prepared. In a separate reaction tube, 10 µL of appropriately diluted enzyme solution, 1.99 mL of the citric acid buffer solution, and 1 mL of the ABTS solution were combined. The contents were mixed thoroughly, and the tube was incubated in a water bath at 30 °C for 3 min. Subsequently, the change in absorbance was measured at 420 nm, with data recorded at 15 s intervals for a total of six data points. The absorbance difference was calculated, and the average value was determined. Enzyme activity was defined as the amount of enzyme required to catalyze 1 µmol of substrate per minute under the specified temperature and pH conditions, corresponding to 1 unit of enzyme activity (U). The formula for the calculation is as follows:

$$U=\frac{∆AV}{\varepsilon 420\cdot v\cdot L\cdot t\cdot C}$$

Here, ΔA denotes the change in absorbance before and after the reaction. V represents the volume of the solution added to the cuvette, measured in liters. ε420 is the extinction coefficient of ABTS, valued at 36,000 L·mol^−1^·cm^−1^. The v variable indicates the volume of the enzyme solution added, expressed in milliliters, while L refers to the path length of the cuvette in centimeters. Additionally, t signifies the reaction time in minutes, and C is equal to 10^−6^ mol.

#### 2.4.2. Optimization of Laccase Production Conditions

The strain was inoculated onto PDA medium and incubated at 28 °C for 30 h to activate its growth. Following activation, holes were punched using a 0.5 cm diameter puncher, and various carbon sources (glucose, starch, fructose, maltose, and sucrose, each at 10 g/L) were added to the basal fermentation medium. Laccase activity was measured by inoculating three fungal disks per flask, with each treatment repeated three times. Cultivation was carried out at 28 °C and 200 rpm for 5 days, and enzyme activity was determined using the ABTS method. The effects of different carbon sources on laccase production were compared to identify the optimal carbon source. Subsequently, the best carbon source was chosen with all other components held constant, and various nitrogen sources (sodium nitrate, potassium nitrate, ammonium sulfate, tryptone, and yeast extract, each at 5 g/L) were evaluated for their effects on enzyme production. Following the selection of the best nitrogen source, the initial pH values of the fermentation medium were adjusted to 4.0, 5.0, 6.0, 7.0, and 8.0, allowing for a comparison of their impacts on laccase production to ascertain the optimal pH value. Building upon the previously established optimal carbon source, nitrogen source, and pH, the optimum cultivation time (48, 72, 96, 120, and 144 h) was further determined.

#### 2.4.3. Laccase Purification

The fermentation broth obtained under optimal conditions was filtered to remove mycelia, resulting in a crude enzyme solution. This solution was subsequently filtered through a 0.22 µm filter. Ammonium sulfate was gradually added to the crude enzyme solution at 4 °C while stirring, until the final concentration reached 60%. After standing for 1 h to allow complete protein precipitation, the mixture was centrifuged at 4 °C and 4200 rpm (1580 g) for 15 min. The supernatant was discarded, and the precipitate was dissolved in 0.02 M sodium acetate buffer (pH 4.8) at a volume one-tenth of that of the crude enzyme solution. The dissolved solution was placed in a 10 kDa molecular weight cutoff dialysis bag and dialyzed against 0.02 M acetate–acetate sodium buffer (pH 4.8) to remove salts. The dialysis solution was replaced every 6 h, and the completion of dialysis was monitored using a 0.1 M BaCl_2_ solution. Dialysis was considered complete when no white precipitate remained in the solution. Upon the completion of dialysis, the enzyme solution was applied to a DEAE-cellulose column with a height of 2.5 cm and a volume of 6 mL. After equilibrating the column with 0.02 M sodium acetate buffer (pH 4.8) for 10 min, the column was washed, and the protein was eluted with 1 M NaCl in 0.02 M sodium acetate buffer. A total of 10 mL of protein solution was collected at a flow rate of 2.5 mL/min. After the protein was electrophoresed on a 15% SDS-PAGE gel, it was stained with 1% Coomassie Brilliant Blue R250 for 15 min and then decolorized for 20 h before imaging. The protein content was determined using the Bradford method [31].

#### 2.4.4. Enzyme Kinetic Parameters

Building on the optimal reaction temperature and pH, various concentrations of the ABTS substrate were prepared to assess the reaction rate. The Michaelis–Menten equation was plotted using the double-reciprocal method, and the kinetic constants *V_m_* and *K_m_* were determined.

#### 2.4.5. Effect of Temperature and pH on the Activity

The purified Tasjk65 enzyme solution was prepared, and reactions were conducted at various temperatures (10, 20, 30, 40, 50, 60, 70, and 80 °C). After incubation for 30 min at each temperature, the enzyme solution was added to the reaction system, and the reaction proceeded at 30 °C for 10 min. Enzyme activity was then measured to evaluate the effect of temperature on laccase activity. Various pH values (3.0, 4.0, 5.0, 6.0, and 7.0, 0.2 mol/L sodium phosphate–0.1 mol/L citrate buffer) were also tested to evaluate their effect on laccase activity.

### 2.5. Degradation of Dyes by Laccase Enzyme

Dye solutions were prepared by dissolving methyl orange (50 mg/L), crystal violet (50 mg/L), malachite green (50 mg/L), neutral red (100 mg/L), and Congo red (1000 mg/L) in 0.02 mol/L of sodium acetate buffer (pH = 4.8). These solutions were then serially diluted to final concentrations of 6 mg/L, 4.5 mg/L, 4.5 mg/L, 9 mg/L, and 300 mg/L, respectively. The reaction system had a total volume of 3 mL. After dilution, the dye solutions (methyl orange, crystal violet, malachite green, neutral red, and Congo red) were mixed with the purified enzyme solution in a 2:1 volume ratio. The reaction was carried out in a thermostatic water bath at 37 °C. After 48 h of degradation at the dye’s maximum absorbance, the absorbance was measured. Methyl orange was measured at 460 nm, neutral red at 550 nm, Congo red at 498 nm, crystal violet at 584 nm, and peacock green at 614 nm. The degradation rate was calculated by comparing the absorbance values between the dye solution in 0.02 mol/L of sodium acetate buffer and the dye solution with the purified laccase enzyme solution.

### 2.6. Genetic and Functional Analysis

#### 2.6.1. Protein Sequence Analysis

After SDS-PAGE, the target band was excised and washed three times with distilled water. Subsequently, 150 μL of a decolorizing solution, prepared by mixing 100 mL of absolute ethanol, 100 mL of acetic acid, and 800 mL of deionized water, was added to decolorize the band. This was followed by four additional washes with distilled water. The gel strip was then sequentially washed twice with 300 μL of 25 mM ammonium bicarbonate, 50% acetonitrile, and 100% acetonitrile to ensure dehydration until the gel block turned white. Following this, 50 μL of 10 mM DTT solution was added, and the mixture was incubated in a water bath at 56 °C for 30 min. Once the temperature had returned to room temperature, an equal volume of 50 mmol/L iodoacetamide (IAM) solution was introduced, and the mixture was alkylated in the dark for 15 min. The gel strip was again washed twice with 300 μL of 25 mM ammonium bicarbonate, 50% acetonitrile, and 100% acetonitrile to ensure dehydration until the gel block turned white. Subsequently, 0.01 µg/µL of proteomics-grade trypsin (20 μL) was added, allowing it to swell on ice until transparent. A 40 μL solution containing 50 mM NH_4_HCO_3_ with 10% acetonitrile was then added to cover the gel. The gel was digested in a water bath at 37 °C overnight. After digestion, the supernatant was transferred to a new EP tube. To the remaining gel block, 100 µL of extraction solution (67% acetonitrile containing 2% formic acid) was added, maintained at 37 °C for 30 min, and then sonicated for 15 min by XinZhi JY92-IIDN Ultrasonic Cell Disruptor (Ningbo XinZhi Biotechnology Co., Ltd., Ningbo, China. With the power set to 10%, working for 5 s and resting for 5 s.). After centrifugation, the supernatants were pooled and concentrated through freeze-drying until completely dry by Thermo Fisher Fresco Freeze Dryer (Thermo Fisher Scientific Shanghai Instruments Co., Ltd., Shanghai, China.). The sample was thus prepared for mass spectrometry analysis.

#### 2.6.2. Sequence Determination Using Mass Spectrometry

Following the centrifugation and drying of the digested peptide samples, they were resolubilized in Nano-LC mobile phase A (0.1% formic acid in water) for sample loading and subsequent online LC-MS analysis. The dissolved samples were loaded onto a nanoViperC18 pre-column (3 μm, 100 Å) in a volume of 2 μL, followed by a 20 μL desalting wash. The liquid chromatography system employed was the Easy nLC1200 nanoliter system (Thermo Fisher, MA, USA), in which the samples were desalted on the pre-column before separation on the analytical column. The analytical column consisted of a C18 reversed-phase chromatography column (75 μm × 25 cm, C18-2 μm, 100 Å. Acclaim PepMap RSLC, Shanghai Xiyun Scientific Instrument Co., Ltd., Shanghai, China.). The gradient used in the experiment involved increasing mobile phase B (80% acetonitrile and 0.1% formic acid) from 5% to 38% over 30 min. Mass spectrometry was conducted using the Thermo Fisher Q Exactive system (Thermo Fisher, USA) in conjunction with a nanoliter spray Nano Flex ion source (Thermo Fisher, USA). The spray voltage was set to 1.9 kV, and the ion transfer tube was heated to 275 °C. The mass spectrometry scanning mode operated in data-dependent acquisition (DDA) mode, targeting up to 20 precursor ions with charge states of 2+ to 5+ for secondary spectrum collection, with the maximum ion injection time for secondary mass spectrometry set to 50 ms. The collision energy (higher-energy collisional dissociation, HCD) was set to 28 eV, applicable to all precursor ions, with dynamic exclusion configured for 25 s.

After obtaining the amino acid sequences through protein sequencing, a comparative analysis was performed on NCBI, followed by the construction of a phylogenetic tree. The phylogenetic tree was constructed using the neighbor-joining method implemented in MEGA version 7.0 (a bootstrap analysis of 1000 replicates was used) using the full-length amino acid sequences of the selected genes. The sequence alignment method is detailed in Section 2.3, and the associated sequence file is provided as the attachment “sequence. fasta”.

#### 2.6.3. Vector Construction and Gene Knockout

**Figure A1:**
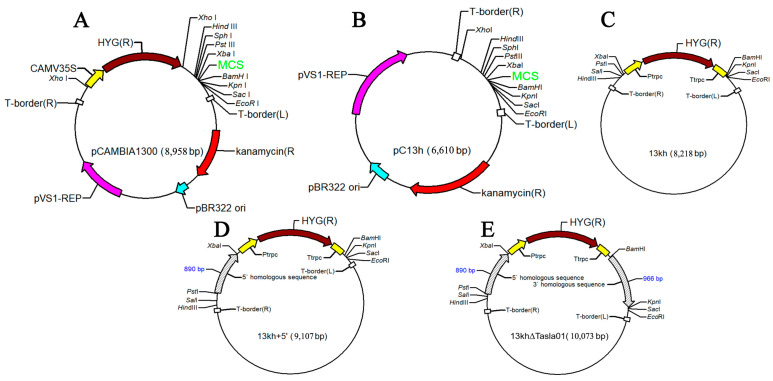
Construction of knockout vector 13kh *∆Tasla01*. (**A**) Vector pCAMBIA1300; (**B**) vector pC13h; (**C**) vector 13kh; (**D**) vector containing an upstream fragment; (**E**) knockout vector 13kh∆Tasla01 (10,073 bp).

The plasmid pCAMBIA1300 (Figure A1A) was digested by *Xho*I and self-ligated by T4 ligase to remove the hph cassette. The resulting plasmid was pC13h (Figure A1B). Subsequently, a 1608 bp fragment containing the promoter regions (Ptrpc, 319 bp), hph ORF (1026 bp), and terminator regions (Ttrpc, 263 bp) was amplified from the plasmid psilent-1 [32]. The *Xba*I and *Bam*HI sites were added to the upper and lower primers, respectively. After digestion with the appropriate restriction enzymes, the fragment was gel-purified and inserted into *Xba*I/*Bam*HI-digested pC13h to produce the vector pC13kh (Figure A1C). The gene knockout vector pC13kh∆Tasla01 was constructed using the pC13kh vector as a backbone, with the main steps outlined as follows: Primers were designed to amplify the upstream fragment of 889 bp (primers PsTasla01SU/XbTasla01SL) and the downstream fragment of 966 bp (primers BaTasla01XU/KpTasla01XL). The upstream fragment was PCR-amplified and purified, and it was subsequently ligated into the 13kh vector through double digestion with PstI/XbaI to produce the vector 13kh+5′ (Figure A1D). After the successful ligation of the downstream fragment, it was PCR-amplified, purified, and then ligated into the knockout vector 13kh∆Tasla01 via double digestion with *Bam*HI/*Kpn*I (Figure A1E).

*Trichoderma conidia* were harvested from the PDA culture and diluted to a concentration of 5 × 10^5^ CFU/mL. This suspension was then mixed with an equal volume of activated *Agrobacterium* AGL1 containing the corresponding vector. After incubation at 28 °C for 24 h on a rotary shaker (220 rpm), the bacterial cells were diluted to OD600 = 0.20, inoculated in induction medium (IM), and cultured at 28 °C and 250 rpm for 6 h. Subsequently, the conidia of Tasjk65 and AGL1 cells were mixed at a ratio of 1:1, and 200 µL of mixture was spread onto a cellophane sheet and placed on an IM plate (90 mm in diameter) containing 0.2 mM acetosyringone. After 48 h of incubation at 23 °C, the cellophane sheet was transferred to a CA plate containing 200 µg/mL cefotaxime and 150 µg/mL hygromycin B and incubated at 25 °C. Each putative mutant was subsequently transferred to PDA medium containing 200 µg/mL cefotaxime and 150 µg/mL hygromycin B. Mitotic stability was tested by subculturing three generations on PDA medium without hygromycin. All primers used in this study are listed in Table A1.

PCR was used to screen the ideal gene knockout transformants. 1. The PCR results obtained with the primers hphU/hphL are positive, indicating that the HYG gene has been successfully integrated into the genome of the strain. 2. The PCR results obtained with the primers Tasla01U/Tasla01L are negative, confirming that the Tasla01 gene has been knocked out. 3. The PCR results obtained with the primers Yzla01U/hphL are positive, indicating that the HYG cassette was inserted at the correct site. 4. The primers 13rbU/hphL were used to confirm the absence of any additional T-DNA insertion during gene disruption, and the result was negative.

### 2.7. Statistical Analysis

All data were analyzed using a one-way analysis of variance (ANOVA) in the R programming language (R version 4.3.1). Statistical differences were established with a *p*-value ≤ 0.05 (variations in figures are differentiated using the letters a, b, c, etc.). All data are presented as mean ± standard deviation (mean ± S.D.).

## 3. Results

### 3.1. Identification of Laccase-Producing Trichoderma Strain

**Figure 1:**
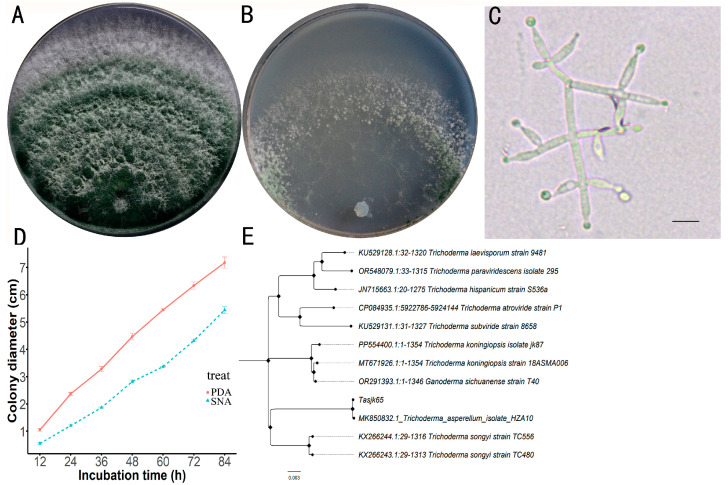
Identification of Tasjk65. (**A**) The morphology on PDA medium. (**B**) The morphology on SNA medium. (**C**) Conidiophores and phialides, with a scale of 10 µm. (**D**) Growth rate. (**E**) Phylogenetic tree.

A total of 117 *Trichoderma* strains were isolated and purified in this study. After screening, the strain with the highest laccase activity was obtained. After activation, the strain was inoculated onto both PDA and SNA media. The Tasjk65 strain formed white colonies on PDA medium during the initial culture. The aerial mycelium was dense, and growth was rapid. After 60 h, conidia began to appear in the inner layer of the colony, and, after 84 h, the colony diameter reached 7.18 cm (Figure 1A). On SNA medium, due to relatively limited nutrients, colony growth was slower. Conidia began to appear after 72 h, and, after 84 h, the colony diameter was 5.44 cm (Figure 1B). The conidia were not clearly segmented, with branches often arranged oppositely, forming acute or nearly right angles. The conidiophores exhibited a symmetrical distribution, and the branching tips formed small stalks resembling bottle shapes, with the conidiophore stalks having an ampulliform shape and a swollen middle section (Figure 1C). The strain exhibited rapid growth on PDA medium, resulting in a substantial production of spores that filled the plate within four days. In contrast, the growth rate on SNA medium was slower, with both the spore production rate and yield being lower than those observed on PDA medium (Figure 1D, raw data see Supplementary Material Data S1). Based on the above cultural characteristics and morphological traits, the strain was preliminarily identified as *T. asperellum*.

After amplifying the *tef1* gene of the strain (NCBI accession number OR865875), NCBI BLAST analysis revealed that the strain exhibited the highest sequence similarity (99.8%) to *T. asperellum* (MK850832). A phylogenetic tree was constructed, as shown in Figure 1E (raw data see Supplementary Material Data S2), in which the tested strain clustered with *T. asperellum* on the same branch. Based on the morphological analysis, the strain was ultimately identified as *T. asperellum* and named Tasjk65.

### 3.2. The Optimal Culture Conditions for the Production of Laccase Enzyme

**Figure 2:**
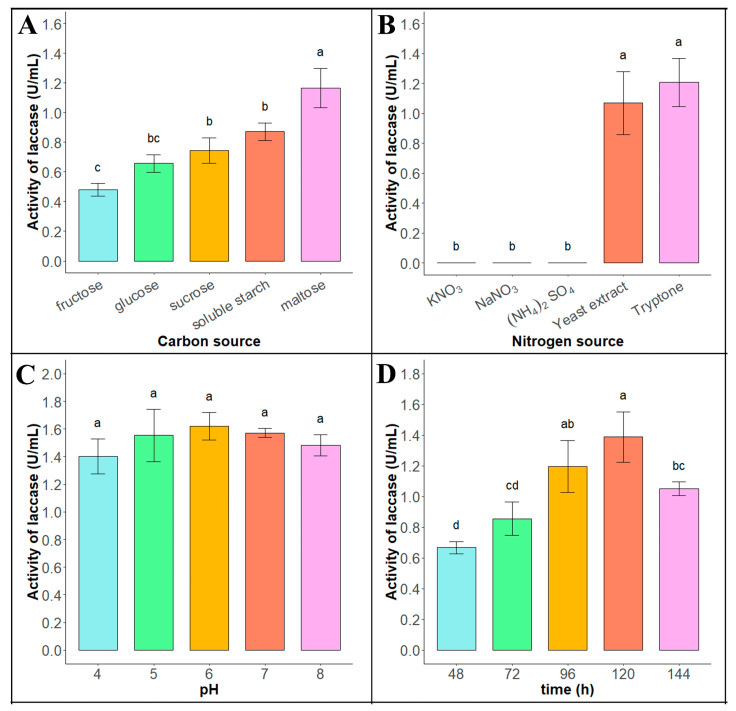
Optimization of enzyme production conditions of Tasjk65. (**A**) Carbon sources. (**B**) Nitrogen sources. (**C**) Initial pH. (**D**) Fermentation time. Statistical analysis was conducted using ANOVA in R. Different letters indicate statistically significant differences (*p* < 0.05), whereas identical letters denote no significant difference.

As shown in Figure 2A (raw data see Supplementary Material Data S3), the enzyme production efficiency of the Tasjk65 strain was higher in fermentation media containing maltose, sucrose, or starch as the carbon source, with the highest enzyme activity observed when maltose (1.166 ± 0.132 U/mL) was used as the carbon source. Therefore, maltose was selected as the optimal carbon source for Tasjk65. As shown in Figure 2B (raw data see Supplementary Material Data S4), the Tasjk65 strain exhibited laccase activity only in the basic fermentation medium containing tryptone (1.207 ± 0.161 U/mL) or yeast extract (1.068 ± 0.21 U/mL) as the nitrogen source, with the highest laccase activity observed when tryptone was used as the nitrogen source. Therefore, tryptone was selected as the optimal nitrogen source for Tasjk65. As shown in Figure 2C (raw data see Supplementary Material Data S5), both acidic and alkaline conditions affected the enzyme production ability of the Tasjk65 strain. The highest enzyme production was observed at pH 6.0 (1.620 ± 0.101 U/mL). Therefore, a pH of 6.0 was selected as the optimal cultivation pH for Tasjk65. As shown in Figure 2D (raw data see Supplementary Material Data S6), the enzyme production of the Tasjk65 strain was related to the inoculation time. Both very short and very long fermentation times negatively affected laccase production. The highest enzyme activity was observed after 120 h (1.32 ± 0.164 U/mL) of cultivation. When the cultivation time was less than 120 h, enzyme production and activity increased. However, after 120 h, both enzyme production and activity decreased. Therefore, 120 h was selected as the optimal cultivation time for Tasjk65.

### 3.3. Purification and Enzymatic Characterization of Laccase from Tasjk65

#### 3.3.1. Laccase Isolation and Purification

The total protein, specific activity, and other data for the laccase enzyme samples and each purification step are shown in Table 1. As can be observed, the total activity of the initial 122 mL crude enzyme solution was 160.78 U, with a total protein content of 1.006 mg and a specific activity of 159.82 U/mg. After ammonium sulfate precipitation, the 10 mL of salt-extracted enzyme solution had a total activity of 44.33 U, a total protein content of 0.161 mg, and a specific activity of 275.34 U/mg, resulting in a purification fold of 1.72 and a recovery rate of 27.6%. After further purification with a DEAE-cellulose ion-exchange column, the total activity of the laccase enzyme solution decreased to 40.32 U, and the total protein content reduced to 0.028 mg. However, the specific activity significantly increased to 1440 U/mg, the purification fold rose to 9.01, and the recovery rate was 25.1%. These results demonstrate a substantial increase in the purification fold of Tasjk65 during the purification process.

#### 3.3.2. Laccase SDS-PAGE Protein Gel Electrophoresis

**Figure 3:**
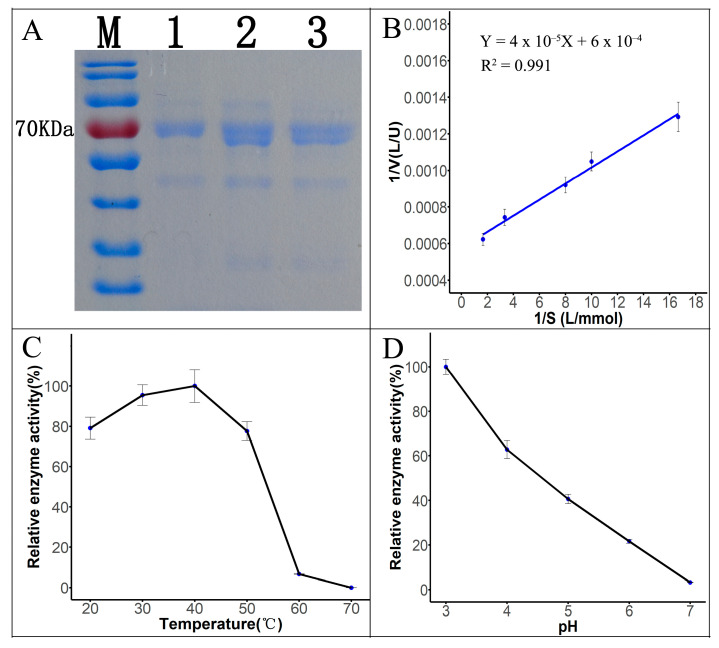
Properties of laccase. (**A**) SDS-PAGE electropherogram. M, marker. Lane1, DEAE-cellulose ion-exchange chromatography. Lane2, ammonium sulfate precipitation. Lane3, crude fermentation broth. (**B**) Lineweaver–Burk plot. (**C**) Effect of temperature on laccase activity. (**D**) Effect of pH on laccase activity.

The fermentation broth of Tasjk65 was filtered through a 0.22 μm filter (Figure 3A, lane 3), subjected to ammonium sulfate precipitation (Figure 3A, lane 2), and further purified using DEAE-cellulose ion-exchange chromatography (Figure 3A, lane 1), followed by SDS-PAGE electrophoresis. The results showed a prominent protein band around 70 kDa. Furthermore, after purification, the number of contaminant proteins gradually decreased (Figure 3A). This indicated that the molecular weight of the laccase produced by this strain was approximately 70 kDa, which is consistent with reported fungal laccase sizes. After ammonium sulfate precipitation and anion-exchange chromatography, the protein was purified 9.01-fold (Table 1). The purified protein was used for subsequent enzymatic kinetic analysis and dye degradation experiments.

#### 3.3.3. Analysis of Enzymatic Properties

*K_m_* is an important parameter of enzymatic properties, reflecting the enzyme’s affinity for the substrate. Using ABTS at different concentrations as the substrate, the reaction rate of the laccase protein was measured at 30 °C and pH 3.0. The *K_m_* value for the catalytic reaction of the ABTS substrate was determined to be 0.06666 mmol/L, with a *V_max_* of 1667 U/L (Figure 3B, raw data see Supplementary Material Data S7), using the Lineweaver–Burk plotting method. Laccase activity was measured at five different temperatures. As shown in Figure 3C (raw data see Supplementary Material Data S8), within the range of 20 °C to 40 °C, the enzyme activity gradually increased with the temperature. The highest laccase activity was observed at 40 °C after 10 min of reaction. At 50 °C to 60 °C, the enzyme activity remained relatively high, but lower than that at 40 °C. After exceeding 60 °C, the enzyme activity sharply decreased. Thus, the optimal reaction temperature for Tasjk65 laccase is around 40 °C, and it shows good heat resistance and strong environmental adaptability. As shown in Figure 3D (raw data see Supplementary Material Data S9), within the pH range of 4.0 to 7.0, the enzyme activity significantly decreased with an increasing pH. The highest activity was observed at pH 3.0, indicating that the optimal pH for laccase produced by Tasjk65 is slightly acidic.

#### 3.3.4. Degradation of Dyes

**Figure 4:**
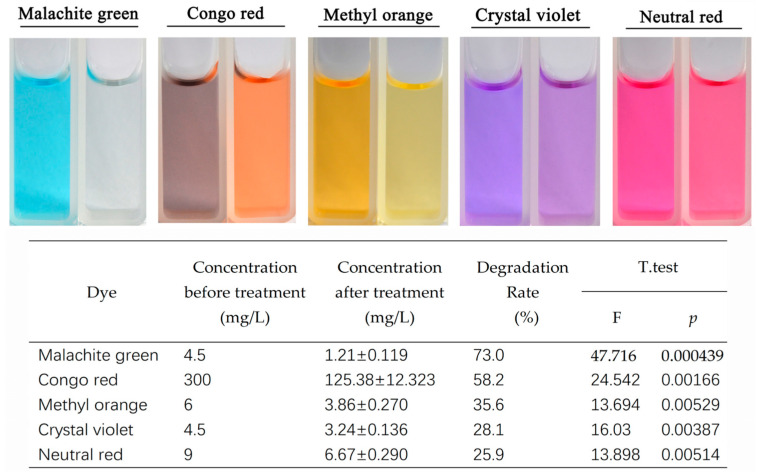
Degradation of dyes by laccase. The left side is the control, and the right side is the treatment. Statistical analysis was conducted using the *t*-test in R, with a *p*-value of less than 0.05 indicating a significant difference.

Laccase exhibited varying degrees of degradation effects on several dyes, such as malachite green, crystal violet, and Congo red (Figure 4). Among these, malachite green showed the highest degradation rate at 73.0%, followed by Congo red with a degradation rate of 58.2%. Degradation effects were also observed for methyl orange, crystal violet, and neutral red, with degradation rates of 35.6%, 28.1%, and 25.9%, respectively. These results indicate that the laccase protein from Tasjk65 can degrade the aforementioned dyes, with it having the most significant effects on malachite green and Congo red. In the absence of any co-solvents, the degradation rate of malachite green exceeded 70% after 48 h.

### 3.4. Cloning of Laccase-Related Genes

**Figure 5:**
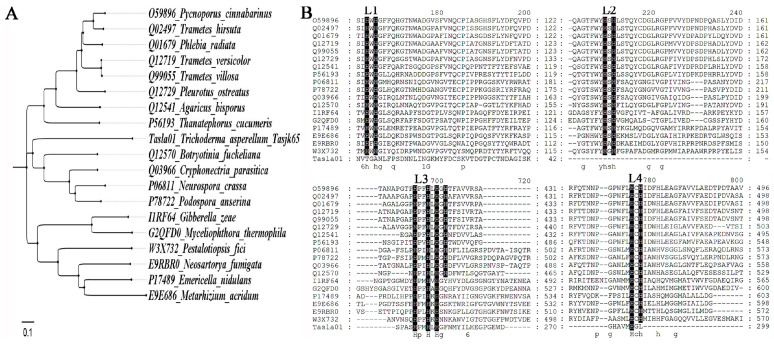
Comparative analysis of laccase-related genes of *Tasla01*. (**A**) Phylogenetic tree of protein of Tasla01. (**B**) Alignment of laccase proteins. L1–L4 represent four conserved regions.

After excising the protein band of approximately 70 kDa and sequencing it, we obtained nine corresponding gene sequences, as presented in Table 2. A comparative analysis (Figure 5A, raw data see Supplementary Material Data S10) revealed that the highest-scoring sequence, namely, Tasla01, belonged to the laccase family of proteins (GenBank accession number PV730304, secondary structure map see Figure S1). Fungal laccases were secreted, and glycosylated proteins contained two to four His-X-His motifs, with the X motif being a cysteine residue that binds copper (Figure 5B, L1–L4). Protein alignment results demonstrated that the *Tasla01* protein exhibited homology with proteins L3. However, motifs 1, 3, and 4 were incomplete.

### 3.5. Functional Analysis of the Tasla01 Gene

#### 3.5.1. Gene Knockout

**Figure 6:**
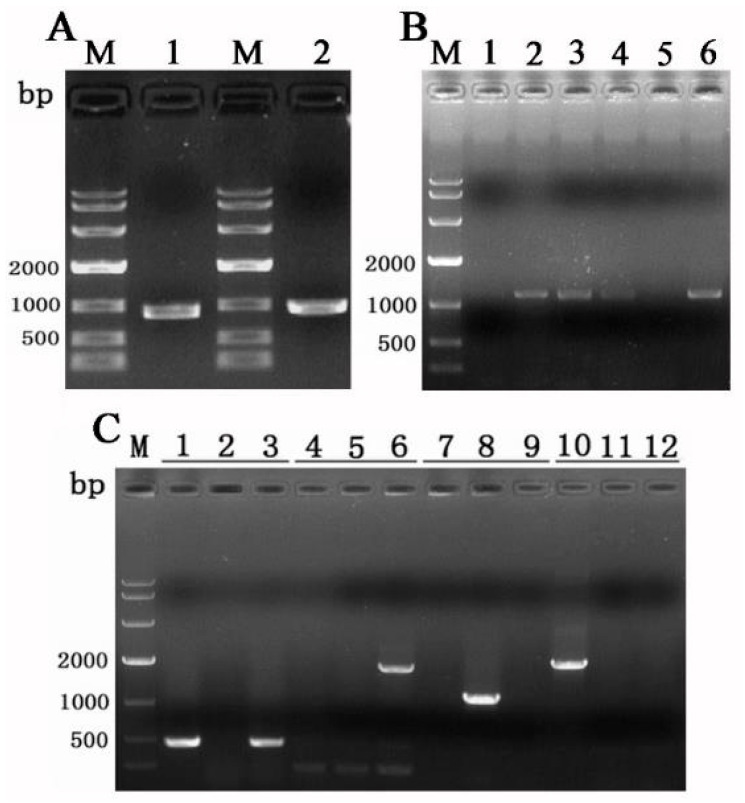
Knockout of *Tasla01* gene. (**A**) Amplification of upstream fragment (1) and downstream fragment (2). (**B**) PCR verification of 6 randomly selected successfully knocked-out mutants. (**C**) Verification of the mutants, where the primers for each set of three are hphU/hphL (lanes 1, 2, 3), Yzla01U/hphL (lanes 4, 5, 6), Tasla01U/Tasla01L (lanes 7, 8, 9), and 13rbU/hphL (lanes 10, 11, 12). The template for samples 1, 4, 7, and 10 is the vector 13kh∆Tasla01; for samples 2, 5, 8, and 11 is Tasjk65 DNA; and for samples 3, 6, 9, and 12 is DNA of *ΔTasla01*.

Gene knockout was conducted using the *Agrobacterium tumefaciens*-mediated transformation (ATMT) method, wherein the upstream fragment of the *Tasla01* gene measured 889 bp, and the downstream fragment measured 966 bp, as illustrated in Figure 6A. A total of 20 co-culture plates were prepared, leading to the selection of 25 potential mutant strains. Following the principle of homologous recombination, the *Tasla01* gene was replaced by the hph sequence in the knockout vector. Subsequently, six mutant strains were randomly selected for DNA extraction, and the Tasla01U/Tasla01L primers were employed to amplify the *Tasla01* gene sequence. The results, depicted in Figure 6B, showed that strains 1 and 5 did not exhibit any bands. Consequently, mutants 1 and 5 were selected for single-spore isolation and purification, followed by DNA extraction. After four rounds of PCR, the ideal *Tasla01* gene knockout mutant was further validated (Figure 6C). The results indicated that the *Tasla01* gene knockout mutants successfully amplified the hph sequence (lane 3). Meanwhile, as hph precisely replaced the *Tasla01* gene, the primers Yzla01U and hphL successfully amplified a band in the upstream homologous sequence of that gene (lane 6). As the *Tasla01* gene had been knocked out, it could not be amplified (lane 9). To eliminate any concern regarding successful gene knockout and the potential random integration of additional T-DNA sequences into other genomic locations that might influence the functionality of other genes, amplification was performed using the T-DNA flanking sequences. The results revealed that the mutants did not amplify the corresponding band (lane 12). Based on the PCR results, we confirmed that the *Tasla01* gene had been successfully knocked out, with no additional T-DNA integration into the genome.

#### 3.5.2. Functional Analysis

**Figure 7:**
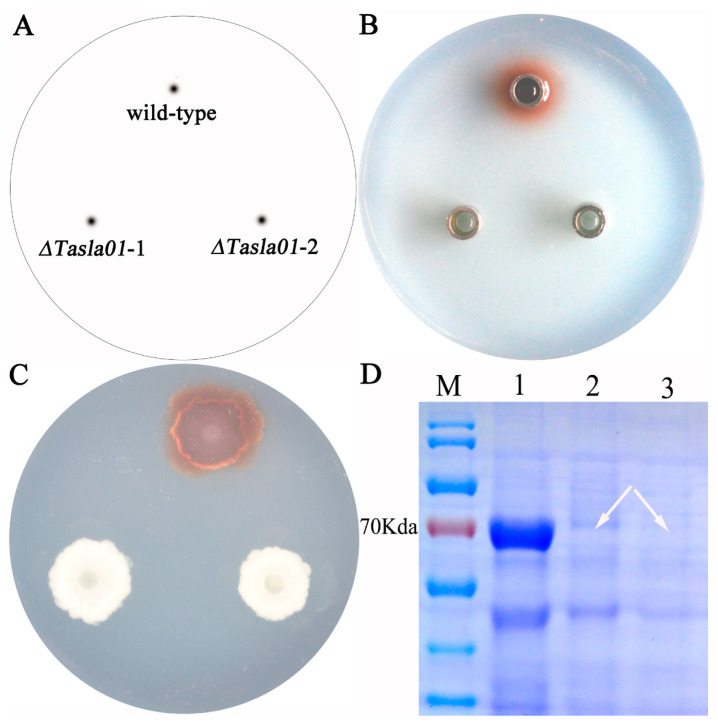
Gene function of *Tasla01*. (**A**) Sample labeling: wild-type is Tasjk65; *ΔTasla01-1* and *ΔTasla01-2* are two random selected knockout mutants of *Tasla01*. (**B**) Oxford Cup Experiment of fermentation liquid on PDA containing guaiacol. (**C**) Enzyme production of strains on PDA containing guaiacol. (**D**) SDS-PAGE electropherogram. M mark. 1, wild-type Tasjk65. 2, *ΔTasla01-1*. 3, *ΔTasla01-2*. The arrow indicates the loss of the protein band.

To further elucidate the function of this gene, we assessed the enzymatic activity of the knockout mutants. The results demonstrated that, following the gene knockout, the strains exhibited no laccase activity. Additionally, when guaiacol (20 microliters per 100 mL of culture medium) was used as a substrate, the wild-type strain produced a red coloration in the culture medium during growth, indicating laccase activity; in contrast, both randomly selected mutant strains did not exhibit any such activity (Figure 7C). The fermentation broth of the wild-type strain also displayed laccase activity, whereas the fermentation broths of the mutant strains exhibited no activity (Figure 7B). SDS-PAGE electrophoresis analysis revealed that knocking out this gene resulted in the disappearance of the target band at approximately 70 kDa, indicating that the gene encodes the protein corresponding to this band (Figure 7D). These findings suggest that the *Tasla01* gene encodes a functional laccase and that its deletion leads to a loss of laccase activity in the strains.

## 4. Analysis and Discussion

Bioremediation technology has gradually become an important option for environmental pollution control due to its efficiency, environmental friendliness, low cost, and wide applicability. As a monomeric protein, the laccase produced by *Trichoderma* species can degrade lignin, participate in melanin synthesis, and degrade environmental pollutants, making it a promising tool in bioremediation. In this study, a laccase-producing strain, Tasjk65, was isolated from soil samples and screened. The cultivation conditions were optimized to improve the laccase production of the strain. The laccase was then purified using various methods, and its enzymatic properties were investigated. The results showed that the laccase produced by Tasjk65 exhibited high enzyme activity at pH 3.0 and around 40 °C, demonstrating strong environmental adaptability. Abd El-Latif AS [20] isolated *T. harzianum* (PP389612) from heavy-metal-contaminated soil and found that it produced an activity of 2.89 U/mL. Umar [33] evaluated the production of laccase by 10 species of *Trichoderma* and found that *T. atroviride* was the highest producer, with an activity of 2.62 U/mL. Mohsen et al. [34] obtained 0.266 U/mL of laccase from *T. viride* after incubation for 96 h at 28 °C using 0.02% guaiacol. Abd El Monssef et al. [2] reported that *T. harzianum* produced 1.286 U/mL of laccase after incubation for 6 days at 30 °C, with an initial pH of 5.5 and the addition of 0.04% guaiacol. Umar et al. [35] evaluated the laccase activity of *T. harzianum* MW785562 by list and found that it was 1.12 ± 0.03 U/mL; additionally, they compared the laccase activities of various *Trichoderma* species, with all results indicating that Tasjk65 has a certain advantage in laccase production.

Moreover, the enzyme showed significant degradation effects on five common organic dyes, with malachite green, Congo red, and methyl orange having the highest degradation rates at 73%, 58.2%, and 35.6%, respectively. Gutiérrez-Soto G [36] found that *Coriolopsis gallica* (Cg) and three laccase isoforms, namely, ThIa, ThIb, and ThII, exhibited degradation rates for crystal violet of 25.0%, 14.3%, 4.3%, and 12.7%, respectively. These results indicate that Tasjk65 has a high degradation efficiency in a short time without any added co-solvents, highlighting its significant potential in textile dye degradation. In practical dye wastewater treatment, a higher bacterial inoculum and longer reaction times increase costs. Therefore, a lower inoculum and efficient degradation make the strain more feasible for industrial application. Future research will focus on investigating the impact of different co-solvents on the dye degradation efficiency of *Trichoderma asperellum* Tasjk65 to enhance its industrial application value.

Laccases derived from fungi represent a significant class of enzymes, typically characterized by a molecular weight of approximately 60–70 kDa and primarily existing as extracellular glycoproteins. The glycosylation content of these enzymes generally ranges from 10% to 25%, underscoring important attributes related to their functionality and stability [37]. Our study demonstrates that the Tasla01 protein consists of 300 amino acids. However, the protein band migrates at approximately 70 kDa, suggesting that the protein undergoes glycosylation. Future research will focus on further elucidating its glycosylation process. Fungal laccases predominantly exist in a monomeric form, with polypeptide chains that fold into three main structural domains, designated as D1, D2, and D3 [38]. Importantly, the functional unit of fungal laccases comprises four catalytic copper atoms that are covalently bonded to the protein backbone via ten histidine residues and one cysteine residue [39]. In our analysis of the laccase produced by Tasjk65, we discovered that motifs 1, 3, and 4 within its structure were incomplete and that it displayed considerable glycosylation modifications. These findings may offer new insights into the mechanisms underlying the enzyme’s activity and stability, thereby providing a foundation for future functional studies. To further investigate the function of this gene, we performed a gene knockout. The knockout mutant and the wild-type strain were inoculated simultaneously on PDA plates containing guaiacol. It was found that the knockout mutant did not produce reddish-brown oxidation rings on the plate, whereas the wild-type strain did. Moreover, the growth of the knockout mutant was not restricted, which differs from the findings of Wu Qiaoyun et al. [40], who observed that both *Mr-lac3* and *Mr-lcc2* knockout mutants showed significantly reduced growth rates on pectin-containing plates compared to the wild-type strain. Furthermore, the NCBI alignment results indicate that protein Tasla01 shares a high degree of similarity with laccase identified by other researchers. However, in comparison to common laccases, the structure of L1–L4 exhibits significant differences. Subsequent studies will focus on whether the phenotypes of the Tasjk65 knockout mutants are influenced by these factors. If a correlation is established, a detailed investigation into the underlying mechanisms will be conducted. This will provide insights into the molecular mechanism of laccase production in the strain, laying a solid theoretical foundation for the industrial application of laccase.

## 5. Conclusions

Laccase is a monomeric protein produced by cells, found both extracellularly and intracellularly. In this study, a high-laccase-producing *Trichoderma* strain, Tasjk65, was isolated and purified from soil obtained in Nanchang, Jiangxi Province. The cultivation conditions of the strain were optimized through single-factor experiments, and the laccase activity of the strain reached 1.32 U/mL. Protein purity was obtained through ammonium sulfate precipitation and ion-exchange chromatography. An enzymatic property analysis revealed that the laccase produced by the strain exhibited high enzyme activity at pH 3.0 and around 40 °C, with a broad pH range and strong adaptability to environmental conditions. Furthermore, the enzyme showed significant degradation effects on five common organic dyes. Simultaneously, the laccase functional gene *Tasla01* was successfully cloned. Although the protein exhibits high homology with other laccases, the distinctiveness of its L1–L4 structure warrants further analysis. This strain holds potential application value for the bioremediation of organic pollutants in the environment. Future research will explore the impact of various factors on the enzyme’s dye degradation efficiency.

## Figures and Tables

**Table 1:** Purification of laccase.

Samples	Volume (mL)	Total Activity (U)	Total Protein (mg)	Specific Activity (U·mg^−1^)	Recovery Rate (%)	Purification Fold
Crude enzyme solution	122	160.78	1.006	159.82	100	1
Purification through ammonium sulfate precipitation and dialysis	10	44.33	0.161	275.34	27.6	1.72
Purification by DEAE-cellulose anion-exchange chromatography	5	40.32	0.028	1440.00	25.1	9.01

**Table 2:** Analysis protein band sequences.

Sequence Number	Genes	Score	Mass (Da)	Matches	emPAI	Number of Introns	Size of mRNA (bp)	Gene Annotation
1	*T* *asla01*	4307	33,002	186 (172)	260.12	2	900	GO:0005507; copper ion binding
2	*T* *asla02*	1814	67,356	34 (33)	2.29	3	1893	GO:0005976; polysaccharide metabolic process
3	*T* *asla03*	424	40,279	11 (10)	1.2	4	1098	GO:0005976; polysaccharide metabolic process
4	*T* *asla04*	493	104,519	20 (18)	0.4	2	2841	GO:0005975; carbohydrate metabolic process
5	*T* *asla05*	302	49,771	11 (10)	0.9	4	1425	GO:0055114; oxidation-reduction process
6	*T* *asla06*	298	52,682	11 (10)	0.83	2	1446	GO:0019050; biological process
7	*T* *asla07*	256	53,629	7 (7)	0.52	0	1476	GO:0004553; hydrolase activity
8	*T* *asla08*	246	62,771	7 (7)	0.43	2	1734	GO:0016884; carbon–nitrogen ligase activity
9	*T* *asla* *09*	150	93,587	4 (4)	0.15	2	2649	GO:0016020; membrane

Notes: Score: The sum of peptide scores reflects the overall confidence in protein identification; a higher score indicates greater reliability. Mass: Protein molecular weight. Matches: Reflects the degree of correspondence between the molecular characteristics of the target protein and those in the protein mass spectrometry profiles of the reference database; a higher score indicates a stronger match. emPAI: Exponentially modified protein abundance index, an indicator of quantitative protein abundance; a higher abundance value generally correlates with a greater concentration of the protein in the sample.

## Data Availability

Data are available from the authors upon reasonable request.

## Supplementary Materials

The following supporting information can be downloaded at: https://www.mdpi.com/article/10.3390/biology14060691/s1, Includes all raw data and sequence information utilized in the experiment. Data S1–S10, raw data of the figures; Figure S1. Secondary structure map of Tasla01 protein.

## Appendix A

**Table A1:** Sequence of primers.

Primers	Sequence (5′-3′)	Size (bp)	Note
Tef1F	CATCGAGAAGTTCGAGAAGG	1366	Identification of strain.
Tef1R	AACTTGCAGGCAATGTGG		
Tasla01U	ATGAGCAATGTGACTGGG	1169	Amplification of ORF of Tasla01.
Tasla01L	TTACAAGCCACTATCAAT		
PsTasla01SU	GGCTGCAGGTTTGCTCGTAGACTGGA	889	Amplification of the upstream homologous arm of the Tasla01 (*Pst*I/*Xba*I).
XbTasla01SL	GGTCTAGATGTTTTCTTTACCAAGTC		
BaTasla01XU	GGGGATCCGAGGGAGAACTGGTGTAT	966	Amplification of the downstream homologous arm of Tasla01 (*Bam*HI/*Kpn*I).
KpTasla01XL	GGGGTACCTGATGATGTGCTGGAACT		
hphU	TGTCCTGCGGGTAAATAGC	476	Amplification of the hygromycin B resistance gene.
hphL	TTGTTGGAGCCGAAATCC		
13rbU	TAGCATTCGCCATTCAGG		Amplification of the side sequence of the vector.
Yzla01U	ATGATCAGGCAACTGACTT		Verify gene knockout.

**Table A2:** Polymerase chain reaction (PCR) mixture.

Components	Volume (μL)	Final Concentrations
TaKaRa Ex Taq	0.25	1.25 U
10×Ex Taq Buffer (Mg^2+^ plus)	5	2 mmol/L
dNTP Mixture	4	0.25 mmol/L
Template DNA	0.5	100 ng
Forward primer	0.5	1 μmol/L
Reverse primer	0.5	1 μmol/L
ddH_2_O	22.5	--

**Table A3:** PCR procedure.

Step	Temperature	Time	Note
1 Pre-denaturation	94 °C	4 min	step2–4 32 cycles
2 Denaturation	94 °C	30 s	
3 Annealing	59 °C	30 s	
4 Extension	72 °C	1 kb/min	
5 Extension	72 °C	5 min	
	10 °C	--	
