# Supplementary figures and images for: Isolation and Characterization of Laccase from Trichoderma asperellum Tasjk65
Source: Biology (Basel). 2025 Jun 13;14(6):691. doi: 10.3390/biology14060691 (PMC12189350; doi:10.3390/biology14060691)

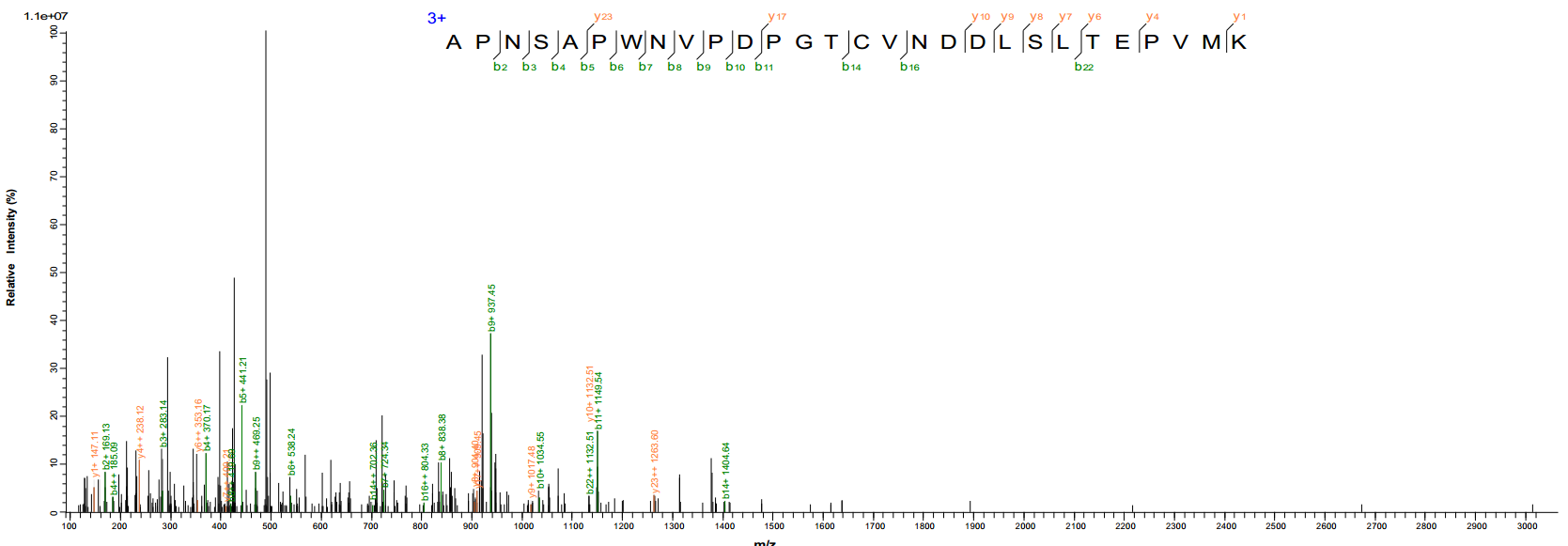

Supplement: Supplementary file 1 [file biology-14-00691-s001.zip › The secondary structure spectrum of the Tasla01.tif]
